# Self-organized Learning from Synthetic and Real-World Data for a Humanoid Exercise Robot

**DOI:** 10.3389/frobt.2022.669719

**Published:** 2022-10-07

**Authors:** Nicolas Duczek, Matthias Kerzel, Philipp Allgeuer, Stefan Wermter

**Affiliations:** Knowledge Technology, Department of Informatics, University of Hamburg, Hamburg, Germany

**Keywords:** self-organizing networks, physical exercise, human-robot interaction, unsupervised learning, synthetic data

## Abstract

We propose a neural learning approach for a humanoid exercise robot that can automatically analyze and correct physical exercises. Such an exercise robot should be able to train many different human partners over time and thus requires the ability for lifelong learning. To this end, we develop a modified Grow-When-Required (GWR) network with recurrent connections, episodic memory and a novel subnode mechanism for learning spatiotemporal relationships of body movements and poses. Once an exercise is successfully demonstrated, the information of pose and movement per frame is stored in the Subnode-GWR network. For every frame, the current pose and motion pair is compared against a predicted output of the GWR, allowing for feedback not only on the pose but also on the velocity of the motion. Since both the pose and motion depend on a user’s body morphology, the exercise demonstration by one individual cannot easily be used as a reference for further users. We allow the GWR to grow online with each further demonstration. The subnode mechanism ensures that exercise information for individual humans is stored and retrieved correctly and is not forgotten over time. In the application scenario, a physical exercise is performed in the presence of an expert like a physiotherapist and then used as a reference for a humanoid robot like Pepper to give feedback on further executions of the same exercise. For evaluation, we developed a new synthetic exercise dataset with virtual avatars. We also test our method on real-world data recorded in an office scenario. Overall, we claim that our novel GWR-based architecture can use a learned exercise reference for different body variations through incremental online learning while preventing catastrophic forgetting, enabling an engaging long-term human-robot experience with a humanoid robot.

## 1 Introduction

A lack of physical exercise is directly linked to many health issues including obesity, cardiovascular diseases as well as depression and anxiety (Booth et al., 2011). Physical activities are quintessential for a healthy lifestyle (Fen and Hong, 2009). Performing physical exercises without proper technique, however, can lead to injuries (Gray and Finch, 2015). As a consequence, supervision by a personal trainer or physiotherapist is of importance for people who are unfamiliar with performing physical exercises. Fitness professionals not only prevent injuries by preventing incorrect technique, but also increase the effects of the exercise by pushing clients closer to their limits and thereby increasing the overall exercise intensity (De Lyon et al., 2017). Despite the benefits, for some people booking a personal trainer is not a possibility, or a physiotherapist is not available. Therefore, the question arises whether a humanoid robot can act in a supportive manner for fitness professionals to encourage clients to exercise. In order to provide a basic service, the humanoid robot is required to be able to engage with its user, and correct any mistakes they make while performing the exercises. Thus, the humanoid robot must be able to detect the pose and movement of a user, compare it to a learned exercise recalled from memory, and provide feedback on it if necessary. This is the primary focus of this article and comes with multiple challenges. First of all, the humanoid robot should be able to learn an exercise and its corresponding pose and movement pattern. Therefore, the memory cannot be fixed beforehand and has to be expandable. Secondly, the learned sequence of poses and movements of an exercise provided by the initial training might mismatch with the current user’s body shape. As a consequence, the memory has to be updated continuously for every new user while maintaining all previous information, i.e., while avoiding forgetting. Finally, the humanoid robot has to be able to provide feedback that is valuable and intuitive to understand for the user, i.e., related to the holistic body pose, not just for instance, which joints are a certain distance off.

To tackle these challenges, for one, OpenPose (Cao et al., 2021) is utilized as the pose estimation framework. Secondly, for memory and learning, a Grow-When-Required Network (GWR) (Marsland et al., 2002) with recurrent connections is used. Finally, due to its humanoid form and its tablet as an easy tool for visual feedback, Pepper is selected as the robot. The resulting novelty of this work is twofold. For one, the recurrent variant of the GWR, called Gamma-GWR (Parisi et al., 2017), is extended in order to counteract catastrophic forgetting and to store many different variations of body shapes for a pose. We call this network Subnode-GWR. For evaluation, we created a novel exercise dataset based on virtual avatars with differing body shapes on which we are able to achieve an average accuracy of 88% with robustness against rotation and translation. Finally, we use the architecture together with a humanoid robot in order to lay the foundations for an interactive physical exercise experience.

In summary, the contributions of this paper center around the extension of previous self-organized approaches to obtain the Subnode-GWR architecture and the evaluation thereof on novel synthetic and real-world exercise datasets. The architecture detects and classifies common exercise errors and is put to use in a test human-robot trainer scenario. In comparison to the other analyzed GWR approaches, the Subnode-GWR approach effectively overcomes the issue of catastrophic forgetting, allowing it to be used in a trainer robot scenario with a dynamic set of users.

The rest of the paper is organized as follows. In section 2, an overview of pose trainers and continual learning is given. In sections 3 and 4, the human-robot trainer scenario and Subnode-GWR architecture are described in detail, and evaluated in section 5. A discussion and conclusion follow, where possible future work is also considered.

## 2 Related work

According to Davis et al. (2018) and Mageau and Vallerand (2003) performance improvement and stress reduction are coupled with a positive relationship between an athlete and his coach. Consequently, a negative encounter with a coach decreases motivation (Newsom et al., 2005; Bartholomew et al., 2009). This also holds for pose trainers that can be categorized as ‘Smart Coaches’, which Gámez Díaz et al. (2020) defines as a “set of smart devices to work independently with the objective of helping people to improve in a specific field”. Past studies have found from a human-robot interaction perspective that social-physical exercise with a robot is more engaging and enjoyable than similar interactions without a physical interaction component (Fasola and Mataric, 2012; Fitter et al., 2020). Specifically, in the context of rehabilitation exercises, it was also, for example, observed by Céspedes et al. (2020) that patient improvement can occur faster if a Socially Assistive Robot (SAR) is integrated into the program. This provides a promising foundation for the development of a robotic trainer.

### 2.1 Pose trainer

One can separate pose trainers into two categories: camera-based and sensor-based. Camera-based approaches can either use RGB-D data as input, i.e. color image with depth information, or solely RGB image data. Sensor-based approaches can also be subdivided into those that just use motion sensors and those that use medical systems like electroencephalograms (EEG) and/or electromyography (EMG). Together with support-vector machines (SVM) proposed by Cortes and Vapnik (1995) as a classifier, EEG has been used by Zhang et al. (2014) for a rehabilitation training system which has been improved by Ukita et al. (2015). Using an EMG and an SVM, Lee (2018) classifies between healthy and sick persons for upper body rehabilitation. In order to detect and analyze protective behavior of patients with chronic pain, Wang et al. (2019) use a long short-term memory (LSTM) network, a recurrent neural network originally proposed by Hochreiter and Schmidhuber (1997), that was fed with surface electromyography (sEMG) data in a stand-to-sit-to-stand scenario.

For camera-based methods, many approaches in the health domain make use of the infrared Microsoft Kinect camera. One of its main advantages is its integrated pose estimation. Ukita et al. (2014) binarily classify the pose of 3D skeletons acquired from a Kinect with an SVM as correct or wrong. Pullen and Seffens (2018) and Trejo and Yuan (2018) similarly classify postures in yoga obtained by Kinects. For weight-lifting, Parisi et al. (2015) predict motion patterns with a self-organizing network and compare them with the real-time poses estimated by a Kinect. While the Kinect is easy to use and has built-in pose estimation based on depth information, its estimation is not very accurate in comparison to current deep-learning human pose estimation approaches.

According to Zheng et al. (2020), in general, human pose estimation is split up into 2D and 3D pose estimation. In 2D human pose estimation, key points that correspond to the two-dimensional spatial location of each joint in an image are extracted, whereas in 3D estimation also depth information is retrieved. In a next step, one can distinguish between single-person and multi-person detection in the 2D domain. The two mainly used deep learning methods for single person detection are regression and body part detection (Zheng et al., 2020). In regression approaches, the pipeline takes an image as an input and outputs key points in an end-to-end manner. Therefore, a direct mapping from the input image to the 2D pose is learned. For body part detection, the pipeline consists of two steps. First, for each body part, a heatmap that indicates the probability for a key point to match the individual joint location is created. In a second step, the key points and the corresponding body parts are put in relation to each other, and the overall pose is generated. The shift from traditional approaches in human pose estimation towards deep learning was pushed by Toshev and Szegedy (2014) and their multi-stage convolutional neural network (CNN) regressor DeepPose. Since then, human pose estimation frameworks have improved steadily, and most of today’s best-performing architectures are based on the body part detection approach. In contrast to single person estimation, multi-person pose estimation faces the challenge of having multiple key points for one joint type that have to be matched to the correct person. Therefore, the idea quickly arose to use a person or object detector like YOLO by Redmon et al. (2016) first in order to receive cropped images where just one person is visible and apply one of the single-person methods. However, this comes with a major drawback, since the accuracy of the human pose estimation depends heavily on the performance of the involved person or object detector. As a solution, bottom-up methods have been developed. One of them is called OpenPose by Cao et al. (2021). As an architecture, it consists of two multi-stage CNNs. For preprocessing, a VGG convolutional network, originally developed by Simonyan and Zisserman (2015) extracts the features of the input image. From these feature maps, the first CNN in an OpenPose architecture computes so-called part affinity field maps (PAFs), that indicate the connection between the joints to form the body part. These PAFs, together with the original image features from VGG19, are used in a second CNN to compute the joint locations for each body part. Finally, these heatmaps are used to match the body parts to the correct person in the scene by applying bipartite matching.

In general, convolutional neural networks are a powerful tool for pose estimation, as, e.g. Kamel et al. (2019) show, who designed a convolutional neural network to provide real-time feedback on Tai Chi poses. Liao et al. (2020) propose a framework that gives a metric for quantifying movement performance. They also introduce scoring functions which map the metric into numerical scores of movement quality. To achieve this, a deep neural network is developed, which generates quality scores for input movements. The neural network receives the joint coordinates as its input that is split into multiple individual body parts and their joint coordinates. The input data for each body part is arranged into temporal pyramids, where multiple scaled versions of the movement repetitions are processed with 1D convolutions and concatenated. Then, the concatenated output is fed into a series of LSTM layers in order to model temporal correlations in learned representations. Finally, a linear layer outputs a movement quality score. Another smart coach proposed by Zou et al. (2018) uses the regional multi-person pose estimation (RMPE) framework developed by Fang et al. (2017) to extract poses from video to generate feedback on the physical exercise performance of users. Recently, Ota et al. (2020) verified OpenPose’s reliability and accuracy on motion analysis for bilateral squats. Therefore, we select OpenPose as our framework to use, since we also analyze a variation of squats as described in section 5. Furthermore, it allows the usage of the humanoid robot Pepper with its built-in RGB camera without requiring an additional depth camera, which increases the usability of our approach. However, as previously mentioned in section 1, the problem still arises how to adapt to different body sizes and variations that significantly mismatch with the trained key points. As a solution, we develop an online learning scheme for our architecture, as a form of incremental learning, that allows for adaption to unknown body shapes.

### 2.2 Continual learning

Continual learning, also referred to as lifelong learning, is deeply integrated into the learning of humans, such that they develop their cognitive and sensorimotoric skills based on novel experiences, as well as repetition and transfer of already acquired knowledge over their lifespan (see Parisi et al., 2019 for a review). Herein also lies the main challenge of continual learning: catastrophic forgetting. Catastrophic forgetting describes the process where previously learned tasks or information are overwritten by novel knowledge (McClelland et al., 1995). This issue finds itself also in the human brain, where it is expressed as a stability-plasticity dilemma (Mermillod et al., 2013). The neural structures in brain areas have to be able to change in order to integrate new information while keeping already acquired knowledge intact. This neurosynaptic plasticity is essential for human learning and is at its highest during early development, where the input is dominated by novel sensorimotor experiences (Parisi et al., 2019). While the brain stays plastic over a lifetime, it becomes less prominent over time when stable neural connections have been established (Hensch et al., 1998). The underlying mechanisms for controlling the plasticity and stability are based on the presynaptic and postsynaptic strength, which was discovered by Hebb (1949). As soon as one neuron is excited by an external stimulus, it activates neurons connected to it. The degree of activation depends on the connection’s strength that is updated based on the presynaptic and post-synaptic activity. While Hebbian plasticity is the basis for neurosynaptic adaptation, the complementary learning system (CLS) theory articulated by McClelland et al. (1995) is the scheme that drives the learning and memorization process. The hippocampus acts as an episodic memory that is highly plastic and therefore learns fast. On the other hand, the neocortex learns slowly and, as a consequence, acts as long-term storage for information. In order to store knowledge and counteract catastrophic forgetting, the structure of the neocortex only changes after receiving similar input over a longer time span. Therefore, the hippocampus replays episodic events to the neocortex, which incorporates the knowledge, given repeated activation of similar structures.

It comes with no surprise that these brain mechanisms have been implemented in lifelong machine learning approaches. One basic approach stems from Kohonen (1990) and is called a self-organizing map (SOM). It has fixed structures consisting of nodes that represent neurons in the brain. To each node, a weight is assigned that defines its place in the input space and therefore also in the lattice of the self-organizing map. This lattice is trained by finding the best-matching node with the least distance to an input sample. The weight is updated according to the difference between the input sample and the node’s distance. Also, neighboring node weights that are connected to the best-matching node are updated. As a consequence, the lattice of the self-organizing map deforms until the average distance to all input samples is minimized. However, since self-organizing maps are fixed in their number of nodes and thus in their dimensions, they are not suitable for multitask challenges in the lifelong learning context. Therefore, self-organizing maps have been extended by, e.g., Growing Neural Gases (Fritzke, 1995). They allow for nodes to be deleted and added. The addition of nodes though occurs after a fixed amount of iterations, which forbids a dynamic growth based on the need for new nodes to represent the input space.

Grow-When-Required (GWR) networks by Marsland et al. (2002) overcome this issue by allowing nodes to be added dynamically whenever the best-matching node’s activity is lower than a predefined threshold. While Grow-When-Required networks are able to learn static input, they lack the possibility to store temporal information between the input samples. Therefore, recurrences are introduced in the Gamma-GWR from Parisi et al. (2017) as context vectors that are additionally stored for every node. They are based on the ideas of the Merge SOM architecture proposed by Strickert and Hammer (2005), where context descriptors capture the activity of the self-organizing map for a given time step. As a consequence, the distance function of the Gamma-GWR not only depends on the weights of a node but also on its context, which is based on the activations experienced in previous time steps. Thus, time sequences can be incorporated in the structure of the Gamma-GWR allowing it to learn, e.g. spatiotemporal sequences. There is a caveat, however, that the input sample in every time step has to be unique, since otherwise, nodes link to themselves, which results in a loop for the time sequence. This is not an issue with the Episodic-GWR (Parisi et al., 2018), which directly stores the predecessor of a node and does not allow for a node to be its own predecessor. This leads to possible loss of information for a time sequence, e.g., a physical exercise where a pose has to be held for a longer period of time, which is why we extend this approach with our Subnode-GWR. Parisi et al. (2016) applied an early form of recursive GWRs to a human motion assessment task. While able to perform well with a fixed feedback threshold parameter on single-subject data, it did not extend well to the multi-subject case, as is addressed in this work.

## 3 Human-robot trainer scenario

In our design, the humanoid robot Pepper from Softbank Robotics acts as a motivator and trainer for the user performing a physical exercise. Pepper has been designed for human-robot interaction especially, featuring built-in speech and face recognition through their NAOqi-API. In its head, microphones, speakers and cameras are installed, and it can move on wheels that are integrated into its triangular base with multiple environment sensors for navigation. Pepper is equipped with tactile sensors in its hands and head. Overall, 17 joints can be manipulated for expressive gestures, and visual feedback can be given on its tablet that is mounted on its chest. While Pepper, in its core, is designed as a humanoid robot, it has no explicit gender, which is also expressed in Pepper’s androgynous voice. When using it for different clients, this is advantageous as studies show that persons are biased towards robots expressing a gender (Siegel et al., 2009; Tay et al., 2014).

**FIGURE 1:**
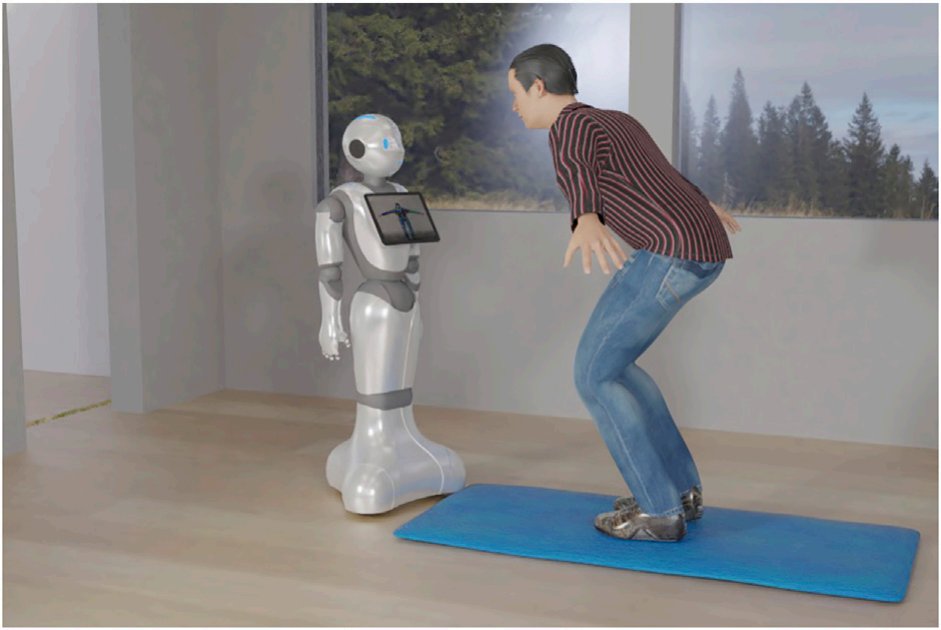
Example scenario, where a user performs physical exercise in front of Pepper, getting feedback via its tablet.

We use Pepper’s tablet to mirror the real-time video feed from Pepper’s front head camera. To do so, the video feed from the camera is streamed to a computer, where it is processed by OpenPose in real-time. The extracted key points are drawn as a skeleton figure on the frame. We also embed the visual feedback into this skeleton figure. Therefore, we compare for each video frame the difference in the joints’ keypoints between the estimation from OpenPose and the target that is inferred from our Subnode-GWR network. If the error is larger than a predefined threshold, we render the corresponding joint in the skeleton as red, indicating that the current joint’s position is wrong. Otherwise, the joint is drawn in green, reflecting the correct positioning of the joint. We stream the frame with the user and the superimposed skeleton figure to a local web server, that can be accessed by Pepper, which is then displayed on its tablet, giving real-time, intuitive and supportive feedback to the user in front of the Pepper robot. The scenario is shown in Figure 1.

**FIGURE 2:**
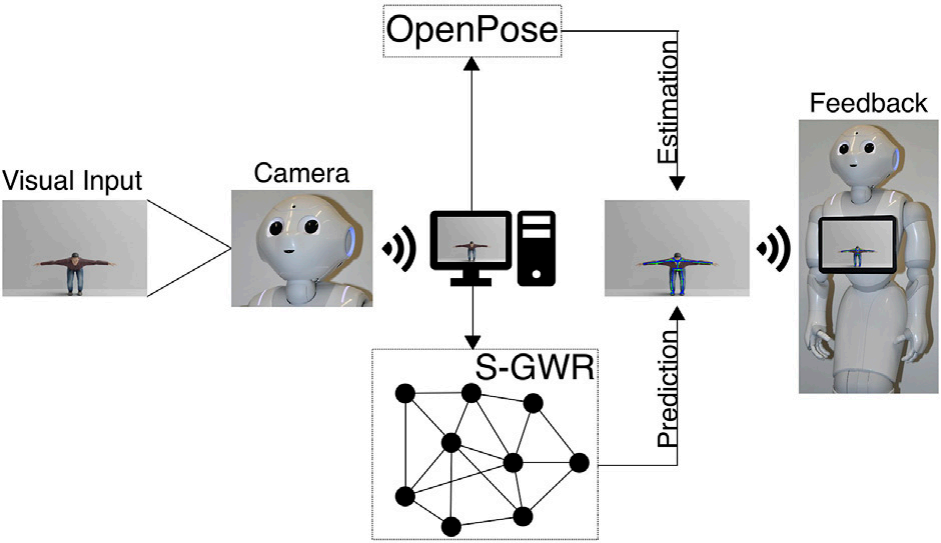
Flowchart that demonstrates the overall scenario with the Pepper and architectures involved.

Additionally, Pepper should react accordingly with verbal and gestural feedback, e.g., praising the user if he/she has performed well, hinting at possible areas of improvement if there is a dominant issue, and motivating the user to continue exercising. For gestural feedback, Pepper’s movement should be restricted to its arms and hands. This is due to the fact, that we record the user in front of Pepper through the integrated camera in its head and need to minimize Pepper’s head movement. In order to correctly process the poses trained on and embedded in the Subnode-GWR, the user is asked by the Pepper to position him-/herself in the camera’s field of view such that no key points are cut off. The real-time estimation by OpenPose is shown on Pepper’s tablet, making it clearly visible to the user whether he/she is positioned accurately. On top of that, we expect that our Subnode-GWR works within a tolerance of 5° in rotation and 5 cm in translation, which we evaluate in section 5. The overall data processing pipeline is illustrated in Figure 2.

## 4 Subnode-GWR

In order to train the Subnode-GWR, a video of a physical exercise that has been performed correctly is processed by OpenPose in order to receive poses as key points for each frame. These key points are normalized according to the image dimensions of a frame and fed into the architecture as the training samples **x**(*t*). The Subnode-GWR is initialized at first with two nodes that are randomly selected from the number of samples. For an input sample, the distance to each node in the Subnode-GWR is calculated as

$$d_{j}=\alpha_{0}{\left\|x\left(t\right)-w_{j}\right\|}_{2}+\overset{K}{\underset{k=1}{\sum }}\alpha_{k}{\left\|C_{k}\left(t\right)-c_{j,k}\right\|}_{2}.$$ (1)

In Eq. 1, **x**(*t*) refers to the sample at time step *t* and **w**_*j*_ to the weight vector of node *j*. **c**_*j*,*k*_ is the context of the *j*th node. It incorporates information of the previous activation in the map up to *k* time steps. **C**_*k*_(*t*) is the context descriptor that is computed as

$$C_{k}\left(t\right)=\beta \cdot w_{b}^{t-1}+\left(1-\beta \right)\cdot c_{b,k-1}^{t-1},$$ (2)

where *β* is a constant that modulates the influence of temporal context, and *b* denotes the best-matching unit (BMU) with the smallest distance of all nodes according to

$$b=\arg\underset{j\in V}{\min}\left(d_{j}\right).$$ (3)

The factors *α*_0_ and *α*_*k*_ are used to balance the influence between the weight vector and the context on the distance to an input sample. In the next step, the activity of the network *a*(*t*) is computed based on the BMU as follows:

$$a\left(t\right)=\exp\left(-d_{b}\right),$$ (4)

which, as a consequence, allows a maximal activity of 1. If the activation *a*(*t*) is lower than a predefined threshold *a*_*t*_, one criteria is met to add a new node to the network. The other criteria is the node’s habituation counter *h*_*j*_ ∈ [0, 1], which allows the nodes to be trained properly, before expanding the network. Being initialized with *h*_*j*_ = 1, each node’s habituation counter is decreased towards 0 over time whenever a BMU has fired. The habituation counter *h*_*b*_ for the BMU and *h*_*n*_ for the neighboring nodes is reduced by

$$\Delta h_{i}=\tau_{i}\cdot \kappa \cdot \left(1-h_{i}\right)-\tau_{i},$$ (5)

where *i* ∈ {*n*, *b*}, and *τ*_*i*_ and *κ* regulate the speed of habituation decrease. According to Parisi et al. (2018), *h*_*b*_ should usually decrease faster than *h*_*n*_, thus *τ*_*b*_ and *τ*_*n*_ are selected such that *τ*_*b*_ > *τ*_*n*_. For the case that *h*_*b*_ as well as *a*(*t*) are less than *h*_*t*_ and *a*_*t*_ respectively, a new node *r* is added to the network by removing the connection between the best-matching and second-best-matching node and connect both to the added node. Its connection age is set to 0. Its weight and context vector are computed as

$$\begin{array}{cc} w_{r} & =\frac{1}{2}\left(x\left(t\right)+w_{b}\right),\\ c_{r,k} & =\frac{1}{2}\left(C_{k}\left(t\right)+c_{b,k}\right). \end{array}$$ (6)

For the case that the activity of the network *a*(*t*) and/or the habituation counter *h*_*b*_ are greater than or equal to the thresholds *a*_*t*_ and/or *h*_*t*_, the BMU *b* and its neighboring nodes are updated as follows

$$\begin{array}{cc} \Delta w_{i} & =\epsilon_{i}\cdot h_{i}\cdot \left(x\left(t\right)-w_{i}\right),\\ \Delta c_{i,k} & =\epsilon_{i}\cdot h_{i}\cdot \left(C_{k}\left(t\right)-c_{i,k}\right), \end{array}$$ (7)

with *i* ∈ {*n*, *b*} and where *ϵ*_*i*_ are constant learning rates that are usually selected as *ϵ*_*b*_ > *ϵ*_*n*_. Also, all connections that end in the BMU *b* are aged by one and will be removed if their age is larger than a predefined threshold *μ*_max_. Finally, all nodes that are not connected to any other node are considered dead and are removed. In contrast to the Episodic-GWR, the information about the successor of a node is not encoded in a matrix *P*, where each connection between nodes is stored and increased by one if two nodes are activated consecutively. While this allows to recall a trajectory of activation by selecting each node’s most frequent consecutively activated BMU, it forbids to select itself as its own successor according to

$$v=\arg\underset{j\in V\backslash i}{\max}P_{\left(i,j\right)}.$$ (8)

Note that up until this point, Eqs. 1–7 are unchanged from Gamma-GWR, and Eqs. 1–8 are unchanged from Episodic-GWR. From here on we modify the architecture for Subnode-GWR. We modify *P*_(*i*,*j*)_ to become $P_{e_{i}}$ , where each row *e*_*i*_ in $P_{e_{i}}$ resembles one physical exercise that the network is supposed to recall. The row itself consists of the best matching units *b*_*i*,*t*_ in consecutive order as they were activated during the last epoch of training on a physical exercise:

$$P_{e_{i}}=\left[\begin{array}{c} e_{0}\\ e_{1}\\ e_{2}\\ \vdots \\ e_{i} \end{array}\right],e_{i}=\left[\begin{array}{c} b_{i,0},b_{i,1},b_{i,2},\ldots ,b_{i,t} \end{array}\right].$$ (9)

There are two advantages to this approach. On the one hand, since Gamma-GWRs solely rely on context to determine a node’s successor, they tend to loop in their prediction if a node references to itself. On the other hand, Episodic-GWRs, according to Eq. 8, forbid nodes to be their own successor at all. This limits the capability of the network to learn physical exercises that require to hold a pose for some frames. These issues are resolved by the modifications described in Eq. 9, which allow for nodes to precede themselves without looping and thus making it possible to store physical exercises, where one pose spans over a longer time frame. The complete algorithm is also depicted in Algorithm 1. After training, the Subnode-GWR can recall the pattern of poses and motion vectors for the trained physical exercise.

Algorithm 1 Training of Subnode-GWR (S-GWR).

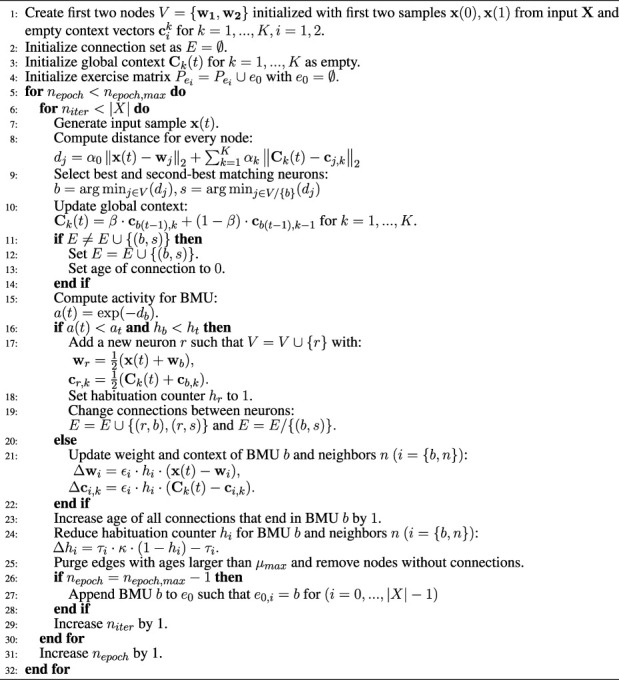

However, the network is tuned for the body dimensions it has been trained on, which limits its ability to be used for analyzing movements from other users. Thus, the primary extension from the Subnode-GWR to the Gamma-GWR stems from the necessity to apply the trajectory of BMUs, that are stored in $P_{e_{i}}$ and resemble a physical exercise, to different body shapes and variations following from mismatching, e.g., age, gender and/or general appearance of the performer of a sample exercise. Hence, we integrate subnodes to the existing nodes. Their weight vector and context vector are computed as:

$$\begin{array}{cc} w_{i,j,l} & =x\left(t\right),\\ c_{i,j,l,k} & =c_{i,j,k}. \end{array}$$ (10)

For a given physical exercise that has previously been trained, i.e., a trajectory *e*_*i*_ exists, we extend each BMU *b*_*i*,*j*_ that currently mismatches the input **x**(*t*) with a subnode. To do so, the weight vector of the current BMU is set for the subnode, as indicated in Eq. 10. Since the entry point to the subnodes is always the parent node, the context *c*_*i*,*j*,*k*_ is simply copied. This allows the Subnode-GWR to easily adapt to new unseen body shapes and variations, while keeping the trajectory of BMUs that maps the physical exercise intact and prevents loss of knowledge about previous body shapes. We use $P_{e_{i}}$ to compare the real-time pose estimation of the user from OpenPose with the weight vector of the current BMU *b*_*i*,*j*_ directly or one of its subnodes *b*_*i*,*j*,*l*_ for exercise *e*_*i*_ if the error on the first frame is lower. The distance between the actual and supposed pose is computed as

$$d_{\mathrm{pose}}={\left\|x\left(t\right)-w_{i,j,l}\right\|}_{2}.$$ (11)

We use *d*_*pose*_ to display the joint-wise error in the current pose compared to the supposed pose of a physical exercise in our human-robot interaction, allowing for precise feedback to the user. Should *d*_*pose*_, however, be larger than a predefined threshold *d*_*t*,*learning*_ on the first frame, the continual learning scheme is triggered, where for each BMU *b*_*i*,*j*_ in trajectory *e*_*i*_ a subnode is created corresponding to the current input pose **x**(*t*). Also, the user is asked to perform the physical exercise once as a baseline. It is important to note that for this step, a fitness professional is advised, since all feedback following is, due to the architecture of the Subnode-GWR, established on this initial performance. Else, if *d*_*pose*_ < *d*_*t*,*learning*_ the training with the Pepper is executed as described beforehand. The algorithm supporting continual learning is shown in Algorithm 2.

Algorithm 2 Continual Learning of Subnode-GWR (S-GWR).

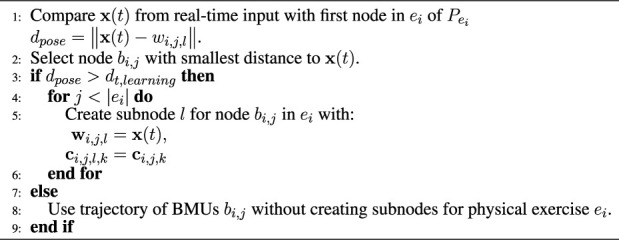

## 5 Experiments on Subnode-GWR performance and robustness

**FIGURE 3:**
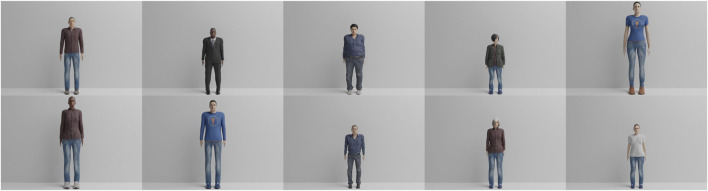
Overview of the ten virtual avatars in the Virtual-Squat dataset, differing in height, weight, clothes, color of skin and gender.

In order to evaluate our approach, two different datasets were created, one synthetic (Virtual-Squat) and one with real-world video recordings (Office-Squat). The synthetic Virtual-Squat dataset^1^ was created using the Blender open-source 3D creation suite^2^ and the MakeHuman open-source creation tool for virtual humans^3^. The dataset consists of ten different avatars (shown in Figure 3). The avatars have randomized heights, weights, body shapes, clothing, skin colors, hairstyles and hair colors to evaluate the robustness of the pose estimation to superficial visual properties of the avatars and the robustness of the exercise analysis to different proportions.

For the physical exercise, we selected a squat where, instead of keeping the arms straight in front of the body as is typical for this exercise, they are raised to the side. The selected exercise offers multiple advantages. First of all, for the user, this movement strains muscles that are required for essential daily activities, e.g. lifting and sitting as well as sports movements (Myer et al., 2014). Secondly, it is challenging for human pose estimation, since all joints are moved. Thirdly, it is a cyclic exercise with two halting points (standing upright, squatting down), which is demanding on the pose prediction. Finally, the camera view is monocular, and the pose estimation is two-dimensional. Therefore, the camera cannot capture the physical exercise extending into the depth while squatting down, increasing the difficulty.

**FIGURE 4:**
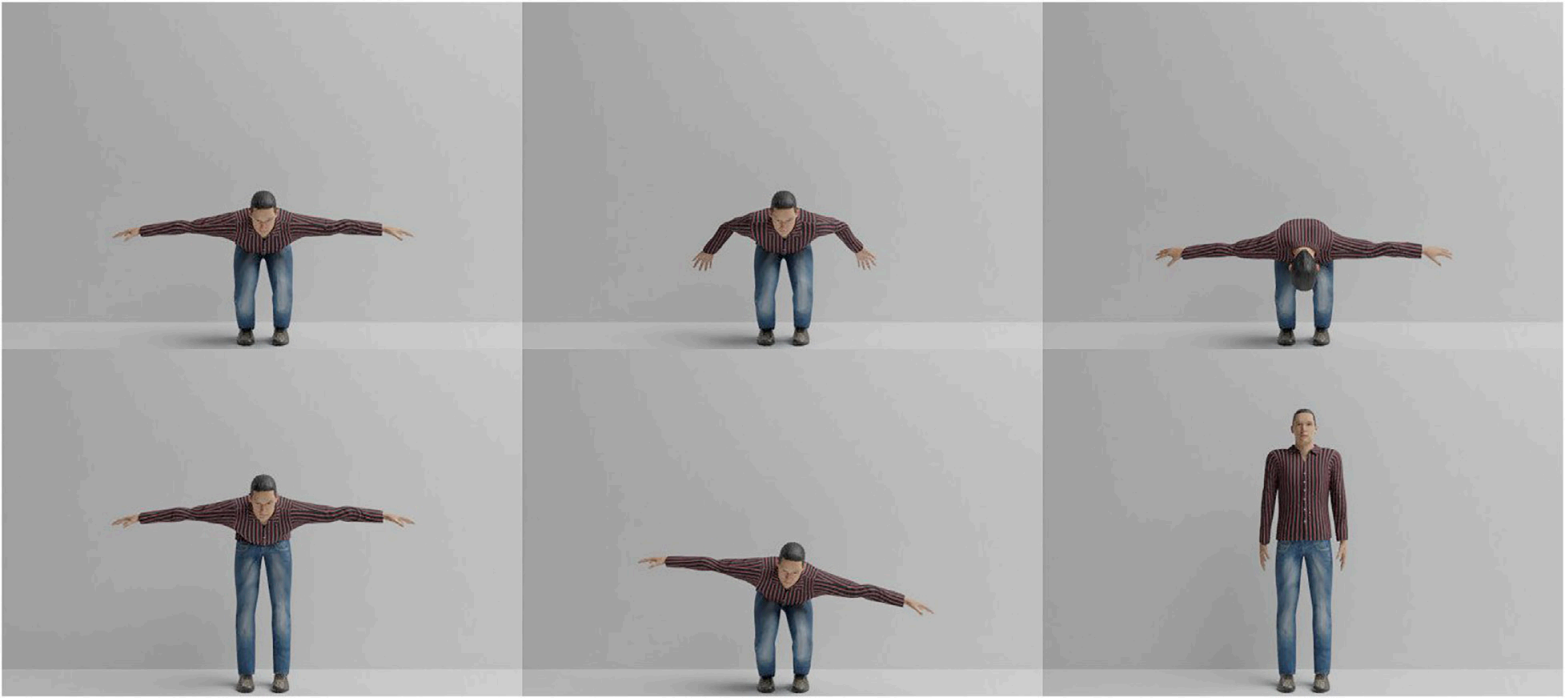
All common errors rendered for virtual avatar one in comparison to the correct execution. Left to right, top to bottom, the illustrated cases are: correct execution, arms not raised, too low, knees not bent, upper body tilt, and too fast. Shown is the 50th frame of the 100-frame videos.

Each avatar repeats the same exercise (squat) in one correct and five incorrect ways. The squat exercise was animated by hand using a video recording of a correctly performed exercise. The five incorrect executions model frequent errors during the exercise. Correct and incorrect exercises, illustrated in Figure 4, were then applied as animations to the ten avatars. Note that the virtual avatars offer the benefit of constructing incorrect exercise executions without burdening real participants with the straining activities. Each exercise is recorded over 100 frames in 480 × 320 resolution using the Cycles rendering engine. Furthermore, to simulate imperfect alignment between Pepper and its interaction partner, each execution of the exercise was recorded in four different ways: 1) with the avatar centered in the image and facing straight ahead, 2) with the avatar rotated by 5° clockwise, 3) with the avatar translated by 5 cm to the left, and 4) with the avatar both rotated and translated. In total, the dataset contains 10 different avatars performing six different exercise executions with four different rotations and translations, leading to 240 exercise videos.

The count of 10 avatars is deemed to be a realistic reflection of the number of participants in a future application that may share an exercise robot at any one time. Variances in appearance, ethnicity, attire and such are dealt with on the level of pose estimation, i.e. by OpenPose, so the number of virtual avatars does not need to be exhaustive in order to produce a robust system. Variances in body morphology are furthermore efficiently handled by the subnode mechanism, which, as previously described, creates a new subnode for participants with significant differences in body size and/or proportions to what has previously been seen. The system scales well with the number of users and thereby subnodes, as the inference time stays constant (same number of nodes), the memory requirement increases slowly and linearly (estimated 150 KB per user), and the prediction accuracy is independent for each subnode. This means that adding further users, especially ones of different body proportions, essentially does not affect the performance of the system for existing users.

**FIGURE 7:**
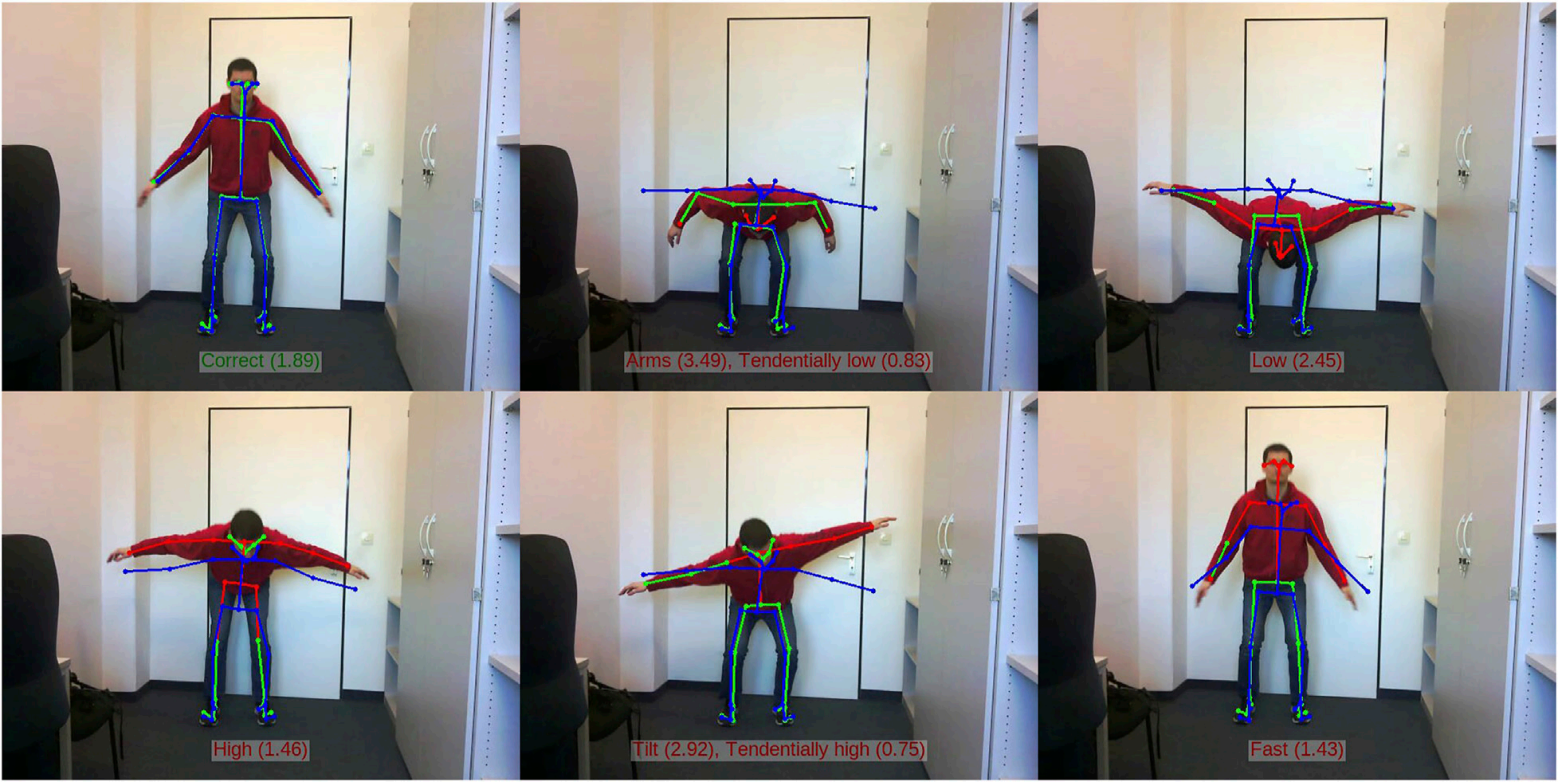
Examples of all common errors in the Office-Squat dataset, overlaid with the pose detection by OpenPose (green-red) and the prediction from the Subnode-GWR (blue). Left to right, top to bottom, the illustrated cases are: correct, arms, low, high (knees not bent), tilt, and fast. If a secondary error classification is predicted that is just under the threshold of being accepted, it is indicated as ‘tendential’. Note that the *fast* case is a snapshot during the ascent phase.

The second dataset created for the purposes of evaluation in this work is the Office-Squat dataset.^4^ This dataset contains 60 videos of 640 × 480 resolution, each showing one execution by the same individual of the same squat as used in the Virtual-Squat dataset. There are 18 correct squat sequences, and 42 with one of the errors shown in Figures 4, 7. Out of the 60 sequences, there are also 13 that intentionally incorporate a translation component, leaving 47 that are similarly aligned. Each video sequence is annotated with a primary and secondary error classification, each out of the list *correct*, *arms*, *low*, *high*, *tilt*, or *fast*. This allows the ground truth for a video to, for example, classify a main error, e.g. *high*, in combination with a secondary error, e.g. *fast*. If there is no secondary error, then the secondary classification is *correct*. Example snapshots from the Office-Squat dataset can be found in Figure 7.

### 5.1 Motion prediction with Gamma-GWR

In our first experiment, we evaluate the Gamma-GWR motion prediction capabilities. We, therefore, process the virtual avatar one squat videos with OpenPose to extract the key points. Then, we train the Gamma-GWR on these key points. It should be noted that all GWR architectures evaluated in this article are nominally trained on only a single video of a correct exercise execution, and then evaluated on other videos from the same avatar or individual. For all architectures, we chose the parameters as noted in Table 1.

**TABLE 1:** Network parameters used for Gamma-GWR, Episodic-GWR and Subnode-GWR used in and optimized on all experiment results.

Parameters	Gamma-GWR	Episodic-GWR	Subnode-GWR
***α***		0.5	
***β***		0.5	
***c***_***k***_	5		1
***ϵ***_***b***_		0.2	
***ϵ***_***i***_		0.001	
***Κ***		1.05	
***τ***_***b***_		0.3	
***τ***_***n***_		0.1	
***a***_***t***_		0.99	
***h***_***t***_		0.3	
***μ***_***age***_		20	
***μ***_***size***_		200	
***d***_***t*,*pose***_		5 pixel (normalized: 0.04)	
***d***_***t*,*learning***_	-		15 pixel (normalized: 0.15)

The Gamma-GWR predicts the successor node *v* by creating a merge vector based on the weight and context of the current BMU *u* comparing it to all node contexts according to

$$\begin{array}{cc} s\left(u\right) & =\arg\underset{v\in V}{\min}\left(d_{s}\left(u,v\right)\right),\\ d_{s}\left(u,v\right) & ={\left\|\mathrm{merge}\left(u\right)-c_{v,k}\right\|}_{2}. \end{array}$$ (12)

**FIGURE 5:**
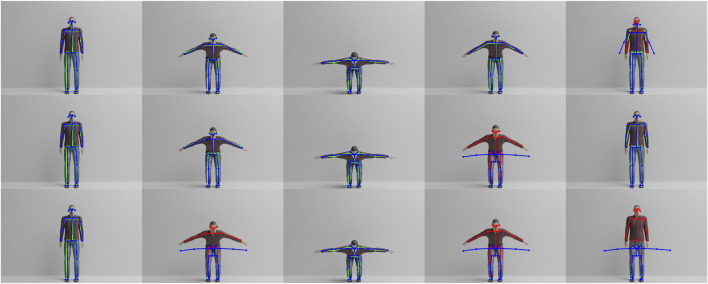
Frames 1, 30, 50, 70 and 100 of avatar one performing the physical exercise. The real-time human pose estimation of OpenPose is shown in green/red and is superposed with a blue skeleton showing the predicted pose from the Gamma-GWR. Red indicates that the mismatch between prediction and real-time estimation is larger than *d*_*t*,*pose*_ for the given joint. The top row corresponds to Gamma-GWR with five predictions, while the middle and bottom rows correspond to 25 and 50 predictions, respectively.

We denote the average joint-wise error over 100 frames per increasing prediction horizon in Table 2. One can see that for the feet the error over all predictions is nearly constant. This is reasonable, since the squat exercise does not involve motion of the feet, which results in near-constant error for the left and right feet. However, for the upper-body joints and face features, the error increases substantially until 50 predictions. After that, it is nearly identical to the results for 100 predictions. To further understand the error for the upper body, we refer to Figure 5 and the corresponding video^5^. From the bottom row of Figure 5, one can see that the architecture is able to process the downward motion but gets stuck in the first halting point and does not recall the upward motion correctly. Therefore, we assume that the Gamma-GWR gets stuck in a loop of a self-referencing node and thus, cannot predict the upward motion. This also explains the similarity between 50 and 100 predictions, since, in both cases, the predictions halt at the same stage of the physical exercise. As a consequence, the question arises whether a mechanism for recalling a trajectory of BMUs as in the Subnode-GWR and the Episodic-GWR, which does not use a prediction scheme based on computation but rather on a look-up table, performs better.

**TABLE 2:** Average joint-wise error in pixels over 100 frames between key point prediction from Gamma-GWR (with increasing number of predicted poses up to 100) and OpenPose’s real-time estimation. Green indicates the smallest error and red the highest.

Gamma-GWR	1	5	10	25	50	100
REye	**1.31**	4.04	12.25	36.04	72.94	**72.95**
LEye	**1.08**	4.11	12.40	36.01	**72.61**	72.60
REar	**2.34**	3.98	10.88	32.63	**65.95**	65.94
LEar	**1.86**	3.49	10.57	32.55	**66.19**	**66.19**
Nose	**0.35**	3.53	11.78	35.31	**71.56**	71.56
Neck	**1.29**	3.20	9.63	27.27	**55.13**	55.12
RShoulder	**0.20**	2.64	9.12	27.51	**55.96**	**55.96**
LShoulder	**1.77**	3.38	9.59	27.66	56.12	**56.13**
RElbow	**0.64**	1.65	6.09	19.39	**39.47**	39.47
LElbow	**0.69**	2.29	6.75	20.41	40.88	**40.88**
RWrist	**1.43**	3.64	9.51	17.96	36.37	**36.39**
LWrist	**1.95**	3.97	9.97	19.54	38.84	**38.84**
MidHip	**2.15**	3.12	5.90	15.00	**28.88**	**28.88**
RHip	**1.73**	2.83	5.74	14.86	28.75	**28.75**
LHip	**1.91**	2.64	5.45	14.49	28.47	**28.47**
RKnee	**2.26**	2.70	3.87	8.56	**15.78**	15.77
LKnee	**1.97**	2.46	3.94	7.91	**14.34**	14.30
RAnkle	2.09	**2.06**	2.06	1.96	**1.87**	**1.87**
LAnkle	**1.43**	1.51	1.83	2.12	**3.02**	3.01
RHeel	**2.74**	2.70	2.61	2.37	**1.97**	2.00
LHeel	**3.46**	3.37	3.33	3.09	2.81	**2.81**
RBigToe	**3.04**	3.03	3.02	2.88	**2.74**	2.74
LBigToe	**3.69**	3.70	3.69	3.74	3.82	**3.82**
RSmallToe	**1.04**	1.06	1.15	1.06	**1.12**	1.11
LSmallToe	**0.99**	1.00	1.09	1.35	1.74	**1.74**
Average	**1.74**	2.88	6.49	16.47	**32.29**	**32.29**

### 5.2 Comparison between GWR variants

In our second experiment, we compare the performance of the Gamma-GWR with five predictions against the Episodic-GWR and our proposed architecture, the Subnode-GWR. For this experiment, we report in Table 3 the average error for all 25 key points over 100 frames between the real-time estimation of OpenPose of the physical exercise performed by the virtual avatar 1 (see upper left image in Figure 3) and the individual prediction method of each architecture.

**TABLE 3:** Average joint-wise error in pixels over 100 frames between key point prediction from Gamma-GWR with five predictions, Episodic-GWR as well as Subnode-GWR and OpenPose’s real-time estimation. Green indicates the smallest error and red the highest.

Avatar 1	Gamma-GWR	Episodic-GWR	Subnode-GWR
REye	**4.04**	2.00	**1.21**
LEye	**4.11**	1.58	**0.85**
REar	**3.98**	2.81	**2.32**
LEar	**3.49**	2.72	**1.94**
Nose	**3.53**	1.80	**0.40**
Neck	**3.20**	1.63	**1.19**
RShoulder	**2.64**	1.54	**0.33**
LShoulder	**3.38**	2.19	**1.73**
RElbow	**1.65**	1.55	**0.71**
LElbow	**2.29**	1.02	**0.61**
RWrist	**3.64**	**1.34**	1.45
LWrist	**3.97**	**1.69**	1.87
MidHip	**3.12**	**1.88**	2.12
RHip	**2.83**	**1.34**	1.68
LHip	**2.64**	**1.78**	1.85
RKnee	**2.70**	**2.12**	2.31
LKnee	**2.46**	**1.75**	1.98
RAnkle	**2.06**	**2.12**	2.10
LAnkle	**1.51**	**1.41**	1.46
RHeel	**2.70**	**2.81**	2.80
LHeel	**3.37**	**3.52**	3.48
RBigToe	**3.03**	**3.05**	3.05
LBigToe	**3.70**	3.71	**3.71**
RSmallToe	**1.06**	**1.04**	1.05
LSmallToe	**1.00**	**0.97**	0.98
Average	**2.88**	1.98	**1.73**

**FIGURE 6:**
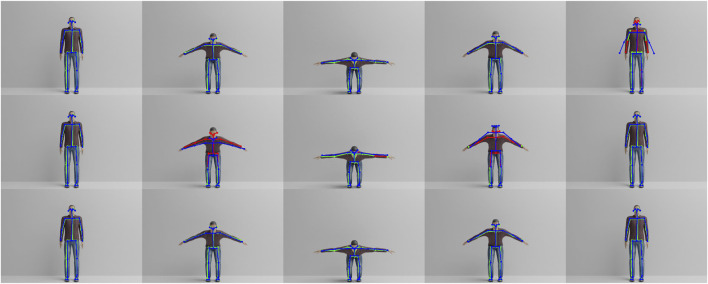
Frames 1, 30, 50, 70 and 100 of avatar one performing the physical exercise. The real-time human pose estimation of OpenPose is shown in green/red and is superposed with a blue skeleton showing the predicted pose from each architecture. Red indicates that the mismatch between prediction and real-time estimation is larger than *d*_*t*,*pose*_ for the given joint. The top row corresponds to 5-prediction Gamma-GWR, while the middle and bottom rows correspond to Episodic-GWR and Subnode-GWR, respectively.

With an average error of 1.73, the Subnode-GWR performs best, with the Episodic-GWR ranking second with 1.98, leaving the Gamma-GWR behind with 2.88. The results show that the prediction algorithm of the Gamma-GWR lacks behind the approach of the Episodic-GWR. In Figure 6, however, it becomes obvious that disallowing nodes to reference themselves leads to asynchronous predictions. Nodes missing in *P* is equivalent to skipping frames in the rendered video. Therefore, the predicted blue skeleton performs the exercise slightly faster than the virtual avatar for Episodic-GWR. This issue is overcome by the Subnode-GWR, which triggers no erroneous feedback, as can be seen in the bottom row of Figure 6, distinguishing the Subnode-GWR as the best approach for the task at hand. Though, while it performs well on virtual avatar 1, on which it was trained, the Subnode-GWR’s novelty lies in its online learning scheme, which is evaluated in a third experiment.

### 5.3 Online learning of subnode-GWR

To test that the Subnode-GWR is able to learn incrementally, we train the network on virtual avatar 1, and then online on the remaining nine virtual avatars. This process involves training the Subnode-GWR on exactly one correct execution video for each virtual avatar in turn, and then evaluating on all videos of all avatars. It can be observed that the performance of the Subnode-GWR does not change at all for any of the avatars when further avatars are trained. This is as expected and is seen to be because further online training can only add subnodes, not modify previous ones, hence preserving the exact performance on previously trained avatars. Note that online learning is required for inference on subsequent avatars as the Subnode-GWR method is intended as one-shot learning, not zero-shot learning. Note also that the inference time, and thereby real-time capability, of the proposed method is dominated by the inference time of OpenPose, which is about 45 ms per 640 × 480 frame (43 ms for the 480 × 320 frames of the virtual dataset) on a relatively modest system with a GTX 1650 GPU and i5 CPU. The inference time of the Subnode-GWR architecture on the same system is approximately 0.8 ms per frame, with peaks of up to 1.3 ms. For reference, this is only 0.1 ms slower on average than the times measured for both Gamma-GWR and Episodic-GWR. A GWR-based approach can have computation time issues when growing beyond a certain size, and this is true for the Subnode-GWR as well. However, the number of nodes in our application scenario grows with the length and number of exercises, not the number of different persons, which are represented by the Subnode mechanism. Therefore, the growing nature of the Subnode-GWR network during incremental learning does not lead to a substantial increase in computation time due to the nature of the subnodes, which have a constant lookup time per node.

Table 4 shows the accuracy result of classifying all joints in all videos (including common error videos) in a binary manner, i.e. as correct or erroneous. For instance, in an incorrectly performed exercise where the arms are in the wrong position, the arm joints are expected to be marked as erroneous, while the remaining joints are expected to be marked as correct, and any deviation thereof by the Subnode-GWR results in a drop in quoted accuracy. For the correct performance of the exercise, the Subnode-GWR is able to give accurate feedback for all joints. As we can see, however, that accuracy reduces to 71.6% for the ‘too low’ error. One can see that for avatar 9 (see Figure 3), the accuracy is substantially lower in comparison to other avatars and common errors. This repeats for the common error where the user performs the exercise too fast. After further investigation, we conclude that this inaccuracy results from the Subnode-GWR selecting the wrong subnode in the first frame. This is due to the fact, that avatar nine resembles many other avatars with nearly matching height and weight features. This leads to the question of how robust the approach overall is against variations in, e.g., rotation and translation.

**TABLE 4:** Accuracy and standard deviation for classifying joints over all avatars performing the exercise including common errors correctly as right or wrong based on *d*_*t*,*pose*_ for a centered position in the field of view of the camera (no rotation or translation).

**Centered**	Correct	Arms	Low	Knees	Tilt	Fast
Avatar 1	1.00	1.00	1.00	1.00	1.00	1.00
Avatar 2	1.00	1.00	0.88	1.00	0.84	1.00
Avatar 3	1.00	0.76	0.92	1.00	0.80	1.00
Avatar 4	1.00	0.92	0.48	1.00	1.00	1.00
Avatar 5	1.00	0.52	0.52	0.96	0.88	1.00
Avatar 6	1.00	1.00	0.60	1.00	1.00	1.00
Avatar 7	1.00	1.00	0.92	1.00	0.96	1.00
Avatar 8	1.00	0.96	1.00	1.00	1.00	1.00
Avatar 9	1.00	1.00	0.36	1.00	0.88	0.08
Avatar 10	1.00	0.32	0.48	1.00	0.92	1.00
Average	1.000	0.848	0.716	0.996	0.928	0.908
Std. Dev	0.000	0.242	0.250	0.013	0.075	0.291

### 5.4 Robustness against rotation and translation

In order to further evaluate the robustness and spot possible drawbacks of the approach, we conduct a fourth experiment, where we rotate each avatar for every exercise by 5°, translate them by 5 cm to the left and lastly combine both rotation and translation. In Table 5, the accuracy for a centered view without rotation and translation is 89.9%, with rotation 88.6%, with translation 90.1% and finally with both rotation and translation combined 83.5%. Overall, the network seems to be unaffected by translation, surprisingly leading to a small increase in accuracy. Rotating the avatars by 5° leads to a small drop in accuracy of around 1%. Combining both rotation and translation reduces the accuracy by around 6%. Most influential to this drop is the common error where the upper body is tilted, which drops by about 15% to an accuracy of 77.2%. Still, reflecting on all common errors in execution and keeping in mind that they have been exaggerated for the avatars in order to properly evaluate robustness on challenging tasks, we feel that with an average accuracy of 88% over all variations and disturbances, the Subnode-GWR is robust against perturbations commonly occurring in the application.

**TABLE 5:** Average accuracy and standard deviation for classifying joints over exercise including common errors with deferring positions (rotation: 5°, translation: 5 cm) correctly as right or wrong based on *d*_*t*,*pose*_.

Variation	Centered	Rotation	Translation	Rot. + Trans
Correct	1.000	0.724	0.980	0.812
Arms	0.848	0.880	0.860	0.720
Low	0.716	0.876	0.752	0.812
Knees	0.996	0.996	0.996	0.988
Tilt	0.928	0.932	0.920	0.772
Fast	0.908	0.908	0.900	0.904
Average	0.899	0.886	0.901	0.835
Std. Dev	0.106	0.091	0.089	0.096

### 5.5 Overall error classification

In order for the Pepper robot to be able to provide more targeted feedback to a prospective user, an expert-knowledge 5-class binary classifier is built on top of the Subnode-GWR output. Given a video sequence as well as the OpenPose detections and Subnode-GWR predictions for it, the classifier generates a score for each of the five error types, and if that score is greater than 1 (the scores are individually multiplicatively scaled so that one is the appropriate threshold for each), this error is predicted to be present. The greater the score, the more confidence there is that the error is severe, allowing the Pepper robot to prioritise which errors to give feedback on and how soon. If greater or less sensitivity to the errors is desired, the threshold of one can be adjusted individually per error class. The predicted primary error is the error with the highest score above 1, or *correct* if no such error exists. The predicted secondary error is similarly defined after excluding the primary error from consideration. Figure 7 shows examples of six different video sequences (video^6^), along with their corresponding classifications and scores as predicted by Subnode-GWR.

**FIGURE 8:**
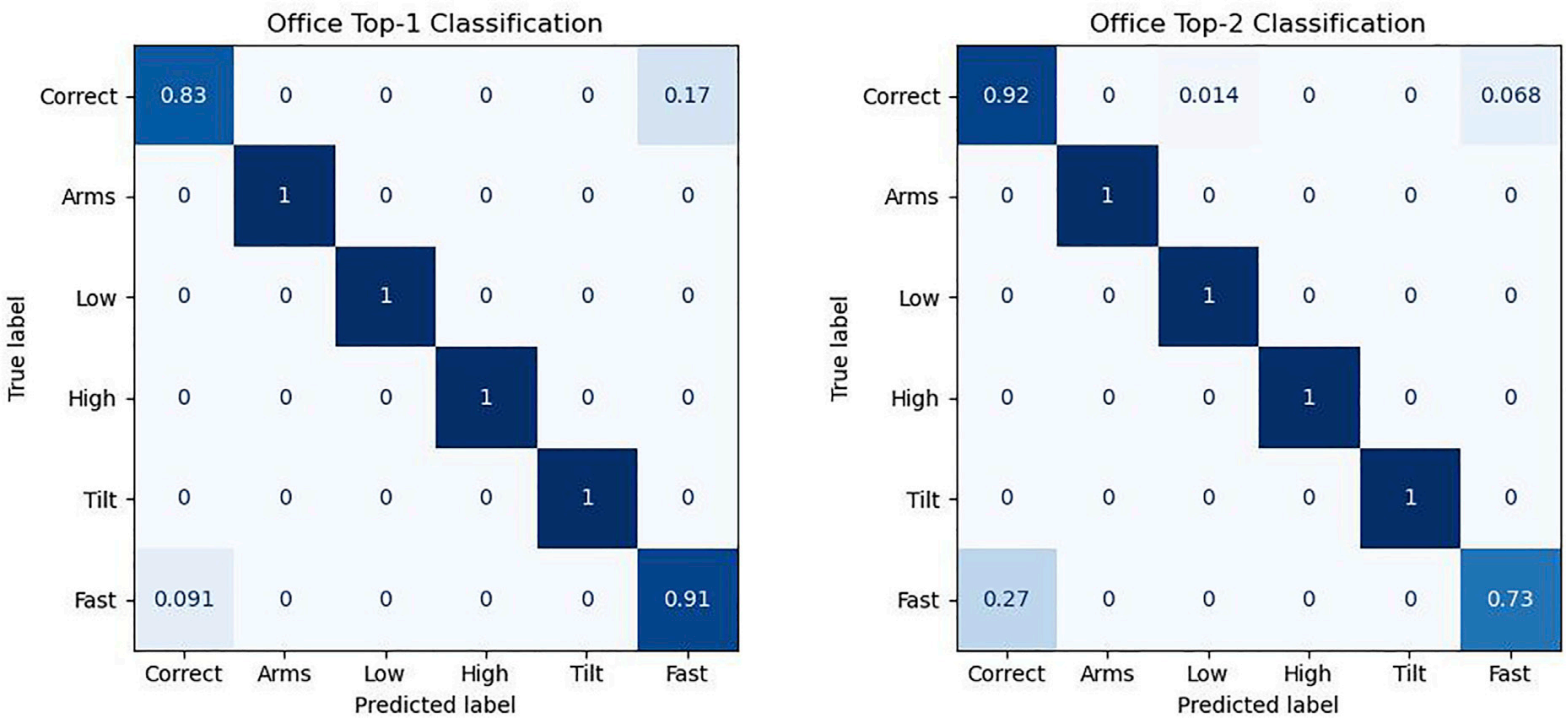
Confusion matrix of the classifier on the Office-Squat dataset for just the primary errors (top-1), and for both the primary and secondary errors (top-2). Many video sequences do not have a secondary error, helping explain why the accuracy of the *correct* class substantially increases for top-2.

The binary classifiers have intentionally been kept very heuristically simple to demonstrate that Subnode-GWR is doing the main work, and are implemented simply by focusing on the mean joint errors of certain joints during certain phases of the video. For example, the *high* score is calculated as the mean upward error in the hip and arm joints during the central third of the exercise, multiplicatively normalized so that one is an appropriate threshold. The *tilt* score, for example, is simply calculated as the (normalized) absolute difference between the mean upward errors in the left and right wrist in the central third of the exercise. Different exercises require different thresholds due to the varying required precision. Therefore we normalize each threshold to a value of one via multiplication. The results of the video sequence classification on the 60 Office-Squat videos are shown in Figure 8. We refer to *top-1* classification if we only compare the predicted primary error with the primary ground truth error, and *top-2* if we compare in an ordered fashion both primary and secondary errors. We observe that all mistakes that the classifier makes relate to the somewhat tricky distinction between the *fast* and *correct* classes (except for a single secondary misclassification of a correct sequence as low). In general, it can be concluded that temporal mistakes are more difficult to identify than visual ones in this scenario. In total, the rate of correct top-1 classification is 93.3% (95.7% if excluding videos with translation), and rate of correct top-2 classification is 85.0% (87.2% without translation).

By comparison, the Virtual-Squat dataset only has single error classification labels, so only the top-1 success rate is relevant, which is 96.7% for no translation/rotation. The only mistakes are two spurious *tilt* classifications for avatar 5, which come about because OpenPose temporarily fails for one arm during the central exercise phase. The addition of translation and rotation to the avatar videos does not change the result other than pure translation, which only misclassifies a single avatar five video, leading to a success rate of 98.3%.

## 6 Discussion

For the most part, a discussion of the core competences and shortcomings of the presented GWR models has been provided along with the previous experimental section. It was seen that episodic memory and context were vital to the performance of the system, especially due to the repeating nature of poses that occur during physical exercises. This alone was not enough, however, to accurately learn the required exercise trajectories, as the ability for temporary pauses in the motion, requiring the same node to remain active over multiple frames, needed to be considered. This was seen to be addressed by the Subnode-GWR approach, which avoided asynchronous predictions and otherwise erroneous feedback to the user due to an obvious mismatch of the predictions.

One clear advantage of Subnode-GWR is that it is able to independently learn the exercise appearance for many user body shapes without adversely mixing and/or forgetting the appearance of previous body shapes. This ability is especially relevant for the continual use of an exercise robot in an environment like a care facility. Our proposed model enables a long-term human robot interaction with many different exercise partners, thus enabling lifelong learning. To cope with many different users, one possible improvement to the method would be to create a new subnode individually for each new user instead of just new users with significantly different proportions. This would lead to a moderate increase in memory use by the model, but not to a level where even thousands of users per robot would become infeasible.

Although the method proved to be robust against mild changes in user orientation and position within the frame, it is surmised that explicit normalization techniques would allow even large translation and scale variations to be dealt with accurately. Such normalization, for example, could be based on the mean dimensions of a human bounding box detection of the user at the beginning phase of each video. Normalization against deviations in user rotation is a significantly more difficult problem, as the 2D-projected trajectory of the human pose keypoints changes in a complex and nonlinear fashion as a function of the rotation angle. One hypothetical solution would be to estimate 3D-human-poses from each video frame sequence and normalize the yaw rotation of these poses before forming a 3D-comparison. The conversion of 2D-video sequences into 3D poses is a notably ambiguous problem, however, as many feasible 3D-poses share the same 2D-projection, even before occlusions are considered.

It is expected in possible future work that the Subnode-GWR architecture could be applied to other tasks without significant overhaul. One example would be gesture recognition, or even hand gesture and/or sign language recognition. Modern pose detection frameworks like OpenPose can estimate finger keypoints in addition to body keypoints, and these could easily be incorporated as additional inputs to the Subnode-GWR. The increased sensitivity of the system to position and orientation changes could be addressed with the aforementioned normalization techniques, with normalization occurring, for instance, relative to the hand bounding boxes. Subnode-GWR could be useful for gesture recognition because it allows aspects like varying body proportions to be dealt with, as well as possibly even allowing adaptation to the slightly varying gesture styles of different individuals. The online learning aspect of Subnode-GWR would also allow gestures to be added or refined on the fly, allowing the system to adapt and evolve dynamically with time.

## 7 Conclusion and future work

Physical exercise is a precondition for a healthy lifestyle, but requires proper technique in order to prevent injuries. To support this, we employed the humanoid robot Pepper as a motivator and feedback giver and developed the GWR algorithm with subnodes and an incremental online learning scheme, which we call Subnode-GWR. While the proposed architecture works well within its purpose, there are still caveats that can be improved. For one, the Subnode-GWR tackles forgetting by increasing the capacity of the network rather than restructuring knowledge. This is, of course, a drawback of the Grow-When-Required approach itself, which has not been solved yet and requires future work. Secondly, the Subnode-GWR requires carefully monitored input from a supervisor (e.g. a physiotherapist) during the learning phase, since its adaptivity is limited within a range of tolerance that has to be tuned manually. Here, future work could improve on the adaption process, making it self-sustained, not requiring additional supervision. Still, we evaluated the Subnode-GWR against already existing GWR variants (Gamma-GWR and Episodic-GWR) and showed the advantages of it. We also examined in further experiments the capabilities of the Subnode-GWR regarding learning on multiple avatars, the robustness against rotation and translation, and the applicability to real-world data. We envision the use of the Subnode-GWR beyond its current application. It can be beneficial in any case where a robust and precise replay of information, e.g., as an episodic memory, is required.

## Data Availability

The datasets used for our studies can be downloaded from: https://www2.informatik.uni-hamburg.de/WTM/corpora/OfficeSquat.zip and https://www2.informatik.uni-hamburg.de/WTM/corpora/VirtualSquat.zip.

## Footnotes

1
https://www2.informatik.uni-hamburg.de/WTM/corpora/VirtualSquat.zip.

2
https://www.blender.org.

3
http://www.makehumancommunity.org.

4
https://www2.informatik.uni-hamburg.de/WTM/corpora/OfficeSquat.zip.

5
https://www2.informatik.uni-hamburg.de/wtm/videos/gwr_virtual.mp4.

6
https://www2.informatik.uni-hamburg.de/wtm/videos/sgwr_office.mp4.
